# Muscle-Specific Tyrosine Kinase-Associated Myasthenia Gravis: A Neuromuscular Surprise

**DOI:** 10.1155/2021/1326442

**Published:** 2021-12-28

**Authors:** Hassam Ali, Rahul Pamarthy, Nayab Ahsan, WashmaAwan, Shiza Sarfraz

**Affiliations:** ^1^Department of Internal Medicine, East Carolina University/Vidant Medical Center, Greenville, NC 27834, USA; ^2^Department of Internal Medicine, Quaid-e-Azam Medical College, Bahawalpur, Punjab 63100, Pakistan; ^3^Department of Internal Medicine, Dow University of Health Sciences, Baba-e-Urdu Road, Karachi, Sindh 74200, Pakistan

## Abstract

Myasthenia gravis is a neuromuscular autoimmune disease that results in skeletal muscle weakness that worsens after periods of activity and improves after rest. Myasthenia gravis means “grave (serious), muscle weakness.” Although not completely curable, it can be managed well with a relatively high quality of life and expectancy. In myasthenia gravis, antibodies against the acetylcholine receptors at the neuromuscular junction interfere with regular muscular contraction. Although most commonly caused by antibodies to the acetylcholine receptor, antibodies against MuSK (muscle-specific kinase) protein can also weaken transmission at the neuromuscular junction. Muscle-specific tyrosine kinase myasthenia gravis (MuSK-Ab MG) is a rare subtype of myasthenia gravis with distinct pathogenesis and unique clinical features. Diagnosis can be challenging due to its atypical presentation as compared to seropositive myasthenia gravis. It responds inconsistently to steroids, but plasma exchange and immunosuppressive therapies have shown promising results. We report a case of a 49-year-old female who presented with acute hypoxic respiratory failure. Our patient experienced progressive, undiagnosed MuSK-Ab MG for years without a diagnosis.

## 1. Introduction

Myasthenia gravis (MG) is an autoimmune disease that affects the neuromuscular junctions of skeletal muscles. The predominant clinical feature is fatigability and weakness that typically become progressively worse during periods of sustained activity and improves after periods of rest [1, 2]. Age of onset of MG is variable with an overall incidence of approximately 15 : 100,000 [1]. Although the majority of patients with MG have antibodies against the acetylcholine receptor (AChR-Ab), 10–15% are seronegative for AChR-Ab. Within this group, about 40% have muscle-specific tyrosine kinase antibodies (MuSK-Ab), representing 5–8% of the MG population [3, 4].

## 2. Case Description

A 49-year-old female presented to the emergency department with worsening respiratory failure. She had a past medical history of significant progressive cachexia, body mass index (BMI) of 14 kg/m2, hypoxic respiratory failure, and progressive diffuse muscular weakness. Additional history revealed progressive symptoms of exertional dyspnea along with intermittent eye drooping and dysphagia for three years. Physical examination was significant for symmetrical generalized gross motor weakness but preserved sensation. Routine initial lab work and imaging were unremarkable. The worsening respiratory failure resulted in intubation and intravenous (IV) steroids for a suspected autoimmune process.

Neurology was consulted who recommended extensive autoimmune workup that was negative (Table 1), and clinical improvement resulted in extubation. Differential diagnoses included autoimmune disease, enzyme deficiency, demyelinating disease, muscular dystrophy, rheumatologic disease, glycogen storage disease, paraneoplastic syndromes, bulbar motor neuron disease, or genetic/mitochondrial myopathies. Imaging was unremarkable except for a small subacute cerebellar stroke. Additionally, thyroid-stimulating hormone (TSH), AM cortisol, complements, and immunoglobulins were normal. An electromyography (EMG) was consistent with a noninflammatory myopathy, and muscle biopsy was negative for myogenic or neurogenic atrophy.

Due to worsening swallowing dysfunction and respiratory and nutritional status, the patient eventually required tracheostomy and percutaneous endoscopic gastrostomy. Plasma exchange was not considered as the diagnosis of myasthenia gravis was missed due to unusual presentation, and the patient could have been in a myasthenic crisis when she underwent intubation. Partial recovery due to empiric steroids resulted insteady improvement of her nutrition and respiratory status. After discharge to inpatient rehab, the myasthenia gravis antibody panel returned positive for MuSK-Ab with a high titer and negative for AChR-Ab. The patient has since been restarted on high-dose prednisone while titrating mycophenolate to a therapeutic dosage. Azathioprine was not selected, although the first line was due to financial issues of the patient.

## 3. Discussion

MuSK-Ab MG is a subcategory of seronegative MG where AChR-Ab is absent. It should be differentiated from double-seronegative myasthenia gravis (dSNMG) that includes patients with MG without detectable antibodies to AChR or MuSK. MuSK-Ab MG patients have shown a female predominance [5]. However, late-onset MuSK-Ab MG shows a slight male prevalence [6]. MG is a highly variable disease with different clinical presentations, including ocular, diaphragmatic or muscular weakness, and sometimes mixed symptoms [7]. Overall, one of the three following presentations are always found in MuSK-Ab MG patients [8],

1. Severe oculobulbar weakness with extreme tongue and facial atrophy
2. Predominant neck, shoulder, and respiratory muscles weakness without ocular symptoms
3. Symptoms similar to AChR-Ab-positive patients.

In MuSK-Ab MG patients, oculobulbar involvement is most common [5, 7], while in seronegative groups, extremity weakness is prevalent [9]. The risk of early generalization of ocular symptoms in seropositive groups is more, while bulbar involvement in seronegative groups [7]. The overall disease course in the MuSK-Ab MG group is pronounced oculobulbar with severe atrophy of muscles in the chronic phase [5]. The pathophysiology of MuSK-Ab MG is related to inhibited postsynaptic clustering of acetylcholine receptors and reduced presynaptic clustering of acetylcholine vesicles [3].

Diagnosis usually involves detecting MuSK antibodies, and this test is usually performed while detecting AChR antibodies. The highly sensitive radioimmunoprecipitation assay (RIA) that uses human recombinant MuSK protein, and detected MuSK-Ab is convenient for the diagnostic indicator of MuSK-Ab MG and also as a mean for monitoring treatment effectiveness [10]. Meticulous clinical and electromyographic criteria must make the diagnosis to rule out other neuromuscular disorders. Inadequate serological testing may miss the genuinely seropositive MG. Abnormalities on single-fiber EMG may be found in MuSK-Ab MG patients [8]. Repetitive nerve stimulation (RNS) testing has low, while single-fiber EMG has high sensitivity in MuSK-Ab MG [5].

The treatment regimen surrounds corticosteroids and immunosuppression; however, a higher corticosteroid dose may be required in higher disease severity [11]. Immunosuppressive agents (prednisone, azathioprine, cyclosporine, mycophenolate, and high-dose cyclophosphamide) are effective in MuSK-Ab MG [9]. Unresponsiveness to anticholinesterases and worsening with standard dosing has been reported in some studies [5, 12]. Cases that are unresponsive to steroids can undergo treatment with a combination of aggressive immunosuppressives and with plasma exchange and IV immunoglobulin [11, 13]. Thymectomy has shown no apparent benefit in MuSK-Ab MG [10, 14].

## 4. Conclusions

Diagnosis of MuSK-Ab MG can be challenging due to its atypical presentation: less prominent symptom fluctuation, predominant respiratory and bulbar symptoms, negative electrodiagnostic studies on limb muscles, and poor or paradoxical response to acetylcholinesterase inhibitors. Our patient suffered from progressive, undiagnosed MuSK-Ab MG for years, culminating in respiratory failure and need for tracheostomy. Our case highlights the challenge of diagnosing MuSK-Ab MG and the importance of having a high clinical suspicion so that early recognition and treatment can help to prevent significant morbidity.

## Figures and Tables

**Table 1:** Autoimmune work up conducted during hospital course.

Autoimmune blood work	Results
Rheumatoid factor (RF)	Negative
Anti-cyclic citrullinated peptide (anti-CCP) antibodies	Negative
Antinuclear antibody (ANA)	Negative
Anti-double-stranded DNA antibody (anti-dsDNA)	Negative
Anti-U1 ribonucleoprotein (RNP) antibody	Negative
Myeloperoxidase (MPO) antibody	Negative
Anti-mitochondrial M2 antibody (AMA-M2)	Negative

## Data Availability

The patient data used to support the findings of this study are restricted by the University and Medical Center IRB, East Carolina University, in order to protect patient privacy. The data are available from Hassam Ali, alih20@ecu.edu, for researchers who meet the criteria for access to confidential data.
